# Do People Become More or Less Materialistic during Disasters? The Mediating Roles of Mortality Salience and Gratitude

**DOI:** 10.3390/ijerph18168566

**Published:** 2021-08-13

**Authors:** Da Jiang, Shuang Liu, John Chi-Kin Lee, Liman Man Wai Li

**Affiliations:** 1 Department of Special Education and Counselling, The Education University of Hong Kong, Tai Po, New Territories, Hong Kong, China; lshuang@eduhk.hk; 2 Integrated Centre for Wellbeing, The Education University of Hong Kong, Tai Po, New Territories, Hong Kong, China; 3 Centre for Psychosocial Health, The Education University of Hong Kong, Tai Po, New Territories, Hong Kong, China; mwli@eduhk.hk; 4 Department of Curriculum and Instruction, The Education University of Hong Kong, Tai Po, New Territories, Hong Kong, China; jcklee@eduhk.hk; 5 Centre for Religious and Spirituality Education, The Education University of Hong Kong, Tai Po, New Territories, Hong Kong, China; 6 Department of Psychology, The Education University of Hong Kong, Tai Po, New Territories, Hong Kong, China

**Keywords:** materialism, mortality salience, gratitude, disaster, COVID-19 pandemic

## Abstract

Studies have yielded inconclusive findings regarding the relationship between disaster experience and materialism. Whereas some have found a positive relationship, others have reported a negative relationship. To clarify the mechanisms underlying these mixed findings, we proposed and examined two mechanisms, namely mortality salience and gratitude. A total of 214 participants (*M*_age_ = 42.05 years, *SD* = 16.49 years) were randomly assigned into either an experimental condition to experience a disaster or a control condition. Participants in the experimental condition reported a lower level of materialism than the scores of their counterparts in the control condition. Such effects were mediated by both mortality salience and gratitude. Participants in the experimental condition reported higher levels of both mortality salience and gratitude simultaneously. Mortality salience strengthened materialism, but gratitude weakened materialism. These findings highlighted the duel-existing mechanisms underlying the relationship between disaster experience and materialism.

## 1. Introduction

The International Federation of Red Cross and Red Crescent Societies (2017) defines a disaster as a sudden, calamitous event with three main features: (1) it interrupts the normal functioning of a community or a society; (2) it causes human, material, and economic or environmental losses; and (3) its impacts exceed the community’s or society’s coping capacity by using its own resources. According to this definition, coronavirus disease (COVID-19) can be categorized as a disaster [1]. In the literature of survival psychology, Sherwood [2] pointed out that individuals change their values and behaviors after experiencing disasters. Because of their devastating disruption to economic and social aspects of life, experiencing disasters may change how individuals see the importance of worldly possession (i.e., their materialism). Materialism is considered a common value orientation, which reflects how one perceives that possessions and material goods are important in one’s life [3]. Interestingly, existing findings show support for both directions of how disasters shape the level of materialism among individuals. Some studies have found that disasters increase individuals’ perceived greater importance on worldly possessions (i.e., a higher level of materialism) [4], while other studies have found that individuals also show an increased intention of donating one’s money possessions to others after a disaster, which reflects a lower level of materialism [5]. These findings may suggest that complex or even opposite underlying mechanisms could be simultaneously observed for the effect of disasters on materialism. To better understand the link between disasters and materialism, we posited two mechanisms underlying the relationship between them, namely mortality salience and gratitude. We hypothesized that a disaster would induce a higher level of mortality salience and that a higher level of mortality salience would be associated with a higher level of materialism. In contrast, we hypothesized that a disaster would also induce more gratitude, which would be associated with a lower level of materialism.

### 1.1. Disasters and Materialism

Disasters influence affective experiences [6,7], decision making [8], and interpersonal preferences [9]. In addition, studies have recently suggested that disasters shift individuals’ views of worldly possessions, such as money (i.e., their materialism) [10], because of their impact on both individual and societal economic status. However, the relationship between disasters and materialism is not fully understood. One line of research has shown that disasters lead to a greater perceived importance on possessions and material goods [4] and greater impulsive consumption, which reflects a high level of materialism [11,12]. For instance, residents who were affected by the Hurricane Katrina crisis reported more impulsive and compulsive buying behaviors [10]. After the 2011 Christchurch earthquake, consumers purchased more utilitarian products [13]. During the ongoing COVID-19 pandemic, consumers around the world have purchased large quantities of daily necessities, resulting in stock shortages around the world, including in China [14], the US [15], and Australia [16]. Materialism has been considered as an attempt to help individuals cope with feelings of personal insecurity and anxiety, and regain control over the situation [17,18,19].

Another line of research has suggested that disasters are associated with a lower level of emphasis on money, such as preferences on donations and prosocial behavior (i.e., donate one’s own money and resources to others). According to Schwartz [20], the behaviors benefiting others are driven by the values of universalism and benevolence that reflect a lower level of materialism [20,21]. Individuals reported charitable donations to disaster victims [22]. Major corporations often donate to disaster relief and reconstruction funds [23,24]. Evidence from a daily survey showed increased donation tendencies during the COVID-19 pandemic [25,26]. In addition, Li et al. [27] examined the relationship between experiencing an earthquake and altruistic giving. In this project, researchers first conducted a survey among children from two rural schools in Sichuan, China, one of the epicenters of the 8 May 2018 earthquake measured at 8.0 on the Richter scale. Researchers then replicated the findings in an experiment in which disaster experiences were manipulated by presenting children with pictures of the earthquake. Across studies, they found that children who experienced an earthquake showed more altruistic giving relative to those who did not. Taking these two lines of research together, little is known about why and how disasters may have contradictory effects on materialism.

Previous work has primarily focused on one mechanism that might be induced by experiencing a disaster, leading to inconsistent findings regarding the relationship between disaster experiences and materialism. Given that a disaster experience may elicit complex thoughts, it is likely that either mechanisms favoring or not favoring materialism can be induced via the disaster experiences. The aim of the current study was to clarify this issue. In particular, we argue that disasters elicit a diverse set of cognitive and affective components. For instance, experiencing a disaster that can cause death may elicit the awareness of the unpredictability of one’s death (i.e., mortality salience). People who have survived a disaster may also feel grateful. Such a feeling of gratitude may be caused by the thoughts that they did not die as some of their counterparts did. It may also be caused by the possibility that they received help and support from others when experiencing the disaster. Although a large number of studies have examined the relationship between experiencing a disaster and mortality salience, and that between experiencing a disaster and gratitude, few studies have considered the relationship between disasters and materialism from a perspective that considers both constructs. Therefore, we aimed to clarify the relationship between disasters and materialism by focusing on two psychological responses to a disaster, namely mortality salience and the feeling of gratitude.

### 1.2. The Relationship between Disasters and Mortality Salience

Disasters foster not only physical threats, but also psychological threats, such as the fear of death [28]. Mortality salience is an awareness of the inevitability and unpredictability of one’s own death [29]. According to the terror management theory (TMT) [30], during disasters, people are strongly aware of death threats, which is at odds with the human instinct to survive. A reaction to the actual or potential threat is critical for survival [2,31,32]. When people perceive the threat level as high, they apply threat reduction strategies to alleviate uncertainty and unpredictability [33,34]. Therefore, disasters would induce mortality salience.

### 1.3. The Relationship between Disasters and Gratitude

In addition to inducing mortality salience, disasters can induce feelings of gratitude [35,36]. Gratitude is a positive emotion that is defined as the recognition that one has benefitted from others’ kindness and appreciation for that kindness [37,38]. Individuals who receive support and benefits are more likely to experience gratitude than those who do not [39]. Socioemotional selectivity theory (SST) [40] posits that people prioritize emotionally meaningful goals (e.g., to experience positive emotion) over knowledge goals (e.g., to learn new knowledge) when they perceive future time as limited (e.g., when they are aware of the fragility of life such as during a disaster) [41]. Searching for emotional meaningfulness has been shown to be important for coping with negative or traumatic events [42]. The feeling of gratitude might be an emotional outcome when individuals search for and reflect on the meaningfulness of life [43].

During a disaster, individuals may feel gratitude for two reasons. First, individuals may receive unanticipated help during a disaster. For instance, governments, communities, and others provide support for affected individuals by constructing and launching economic and health support structures [44], as well as the mass media reports, measures, and behaviors related to governmental, societal, and interpersonal support [36,45]. These kinds of support may induce feelings of gratitude. Second, an important characteristic of gratitude is the tendency to consider all of life as a gift [46]. Individuals who endure and survive a life-threatening event might be less likely to take life for granted and therefore feel more grateful [43]. Indeed, Taylor [47] found that individuals with life-threatening diseases reported a greater appreciation for life. Researchers found that people appreciated their ordinary life and social connectedness to a greater extent after the occurrence of an earthquake [48]. Emmons [49] found that experiencing extreme difficulties in life was a source of trait gratitude. Frias and colleagues [43] posited that when individuals are aware that life is a benefit, not a given, they feel gratitude. Such a feeling may be particularly salient during disasters when individuals may see others suffer and even die. They also empirically demonstrated that life-threatening circumstances enhance the feeling of gratitude [43]. A study found that mortality salience enhanced the appreciation of a meaningful film but only for those participants who revealed a high rating of searching for meaning for life [50]. The study found that, for older Chinese adults, participants in the gratitude induction reported lower death anxiety as compared with those participants with a hassle and a neutral condition [51]. A large number of posts on Twitter expressed the feeling of gratitude in the context of two notable disasters, a mass shooting in Connecticut in 2012 and a tornado in Alabama in 2011 [36].

### 1.4. The Relationship between Mortality Salience and Materialism

Terror management theory [30] posits two mechanisms that buffer anxiety causes by the awareness of the inevitability of death, namely cultural worldview validation and self-esteem enhancement. After a manipulation to induce mortality salience, Greenberg et al. [52] found that people punished those who confront and reward those who confirm with their cultural worldview. Because in the capitalist cultures one common worldview regards wealth and possessions as the path to success and happy life, predictions based on the TMT argued that people who perceive a greater level of mortality salience would attach more importance to worldly possessions [18,53,54].

The positive relationship between mortality salience and materialism has been supported by a number of empirical studies. Kasser and Sheldon [55] found that participants in the mortality salience condition showed a higher level of financial expectations for themselves than their counterparts in the control condition. They further found that participants in the mortality salience condition showed a higher level of greed and consumed more resources to profit more in a game. Arndt et al. [53] argued that thoughts about death triggered individuals to lean on materialism to cope with death-related fear and anxiety. Similarly, studies found that teenagers with less advantageous socioeconomic circumstances reported a higher level of materialism [56]. Longitudinal data assessed from 2005 to 2013 found that individuals reported a higher level of materialism when they were threatened by lower wages and higher unemployment rates due to the housing and financial crisis that happened in America [57]. Because of the relationship between disaster and mortality salience, and that between mortality salience and materialism, we hypothesized mortality salience to mediate the relationship between disasters and materialism.

### 1.5. The Relationship between Gratitude and Materialism

Gratitude is a common positive emotion to help people find relief [38], which can alleviate the stress, anxiety, and depression caused in the context of disasters. For instance, the feeling of gratitude buffers psychological distress and promotes growth in psychological resources after the September 11th attacks [58]. Cancer patients with a higher level of gratitude reported better psychological well-being [59,60,61,62]. Previous studies found that interventions to induce the feeling of gratitude could help older adults and patients reduce anxiety and depressive symptoms and maintain their personal and relational well-being [51,63,64].

Polak and McCullough [65] argued that feelings of gratitude could remind individuals of the goodwill of other people, increase psychological security, and therefore reduce materialistic strivings. Indeed, the trait of gratitude was associated with lower levels of materialism [38]. Similarly, Polak [66] found a negative relationship between gratitude and materialism. An experimental study showed that the self-reported materialism reduced after adolescents recorded gratitude journal entries daily for two weeks [67]. Because of the relationship between disaster and gratitude, and that between gratitude and materialism, we hypothesized gratitude to mediate the relationship between disasters and materialism.

### 1.6. The Situation of COVID-19 and the Current Study

As of 31 December 2020, COVID-19 has affected 219 countries and territories, with more than 85 million cases of infected people, and more than 1.8 million deaths [68]. As COVID-19 has led the global economy into the worst recession since World War II [69] and causes individual harm as an unpredictable and uncontrollable stimulus [70], it has been considered a disaster [71]. Like Severe Acute Respiratory Syndrome (SARS), the Christchurch earthquake, and Hurricane Irma, the COVID-19 pandemic has significantly impacted human behaviors by causing increased unemployment, company failures, and global economic downturns [72]. Moreover, since the fatality risk of COVID-19 is higher than most infectious diseases, the COVID-19 pandemic provides an extreme and complex emotional context in disasters [73]. Therefore, the COVID-19 pandemic could be considered as an appropriate situation to unpack the contradictory relationship between disasters and materialism.

We argue that the inconsistent relationship found between disasters and materialism is attributable to the fact that disaster experience may induce dual psychological systems, namely mortality salience and gratitude. When individuals focus on mortality salience, they would show a higher level of materialism as a way of coping with anxiety. When individuals focus on gratitude, they would show a lower level of materialism because the feeling of gratitude has eased anxiety caused by the disaster. We adopted an experimental design to study the causal relationship between disasters and materialism. In particular, we examined the mediating roles of mortality salience and gratitude in this relationship. We manipulated the disaster experience by asking participants to watch a video of street view in Wuhan, China during the COVID-19 lockdown in 2020. In the non-disaster focus control condition, participants viewed a street view in Hangzhou, China taken in 2018. We hypothesized that participants in the experimental condition would report higher levels of both mortality salience and gratitude than the control condition (*H1*), and that mortality salience would be positively associated with materialism (*H2*), whereas gratitude would be negatively associated with materialism (*H3*).

## 2. Materials and Methods

### 2.1. Participants

A convenient sampling method was adopted in this study. Participants were recruited through university mass mail systems and the public WeChat platform (Tencent, Shenzhen, China). Based on power analysis conducted by G*Power Program before data collection [74], after setting the statistical power to 0.80, with a medium effect size of 0.25 in an ANOVA test with two conditions and three covariates, the target sample size was 196. In total, 214 adults aged from 18 to 81 (*M* = 42.05 years, *SD* = 16.49 years) from 22 provinces in China participated in the study in May 2020. This study was approved by the Institutional Review Board of the Education University of Hong Kong. All of the participants completed an informed consent form. The invalid responses of 21 participants were excluded from data analysis because of the technical problems with the online experiment (e.g., videos could not be properly played due to low-speed Internet connections). The final sample comprised 193 participants (70% females; *M* = 40.23 years, *SD* = 15.59 years).

### 2.2. Procedure

Participants were randomly assigned to either the experimental condition (*n* = 86) or the control condition (*n* = 107). In each condition, participants were asked to watch a video clip with careful attention. They were informed that they would be asked some questions related to the content of the video in the next part of the experiment. After watching the video, the participants were required to recall “a vivid personal experience that was directly or indirectly relevant to the watched video, which could be positive/negative/neutral,” and to provide basic information on the experience in five areas: “When did it happen?”, “Where did it take place?”, “Who was involved?”, “What happened?” and “How did you feel/think?”. They were asked to rate to what extent they could memorize the experiences. Next, the participants were instructed to respond to questions regarding their actual affect and attitudes toward materialism, as well as demographic information. Following these steps, each of the participants received a supermarket coupon worth approximately USD 7.

### 2.3. Experimental Materials

The two videos clips, each lasting for 2 to 3 min, were equally randomly displayed to the participants under the two conditions. The video clip in the experimental condition showed the street view during the lockdown in Wuhan caused by the COVID-19 pandemic. The video showed images of sick persons, the separation of families, and empty roads during the lockdown period in Wuhan. In contrast, the video clip in the control condition presented the street view in Hangzhou (taken in 2018), showing the daily life of healthy people, reunion dinners with friends and family, and a crowded and thriving tourist resort. Similar to Wuhan, Hangzhou is a well-known city and one of the financial centers in China.

As a manipulation check to ensure that the video presented in the experimental condition successfully activated participants with thoughts related to COVID-19, two independent raters read the written description provided by the participants in the two conditions and coded whether the described experiences were relevant to COVID-19 on a binary scale, with 1 = “*Yes*”, 0 = “*No*”. In the experimental condition, 77.91% of the experiences were coded as relevant to COVID-19, whereas only 0.93% of the experiences written by participants in the control condition were coded as relevant to COVID-19, *X*^2^ (1, 193) = 123.79, *p* < 0.001. The results indicated that the manipulation was successful.

### 2.4. Measures

#### 2.4.1. Materialism

We measured materialism using the short form of the Material Values Scale (MVS) as updated by Richins [75]. The scale suggested three dominant subscales to measure materialism: success (the use of possessions to judge the success of others and oneself, e.g., “Some of the most important achievements in life include acquiring possessions”), happiness (possessions and acquisition lead to life satisfaction, e.g., “I’d be happier if I could afford to buy more things”), and centrality (the centrality of possessions in a person’s life, e.g., “I try to keep my life simple, as far as possessions are concerned” (reversed coded)). Participants indicated on a 5-point Likert scale to what extent they agreed or disagreed with the views about the use of possessions to judge success, ranging from 1 “*completely disagree*” to 5 “*completely agree*”. More materialistic individuals tend to place material property at the core of their lives and use their possessions to measure their success and happiness, and consider possessions as an indicator of a central position. As the measure consists of nine items, we used Cronbach’s alpha to assess the scale’s reliability for our participants. In the present study, the internal consistency was acceptable (*α* = 0.73).

#### 2.4.2. Mortality Salience

Participants answered two questions regarding whether and to what extent they had been thinking about death when they watched the video or wrote about their experiences. They used 0 “*no*” or 1 “*yes*” to answer whether they had thought about death. They indicated the extent of the thought on a 7-point Likert scale from 1 “*very weak*” to 7 “*very strong*”. We multiplied the responses of these two questions so that the scores of those who did not think about death when watching the video were coded as 0.

#### 2.4.3. Gratitude

Participants were asked to indicate the intensity of feelings of gratitude when watching the video or writing their experiences on a 5-point Likert scale ranging from 1 “*not at all*” to 5 “*very much*”.

#### 2.4.4. Accuracy of Memories

After recalling relevant personal events, participants were asked to indicate the level of accuracy of their memory on a 5-point scale from 1 “*not at all*” to 5 “*very much*”.

#### 2.4.5. Control Variables

We controlled for participants’ personal income and socioeconomic status [76] because they were found to be associated with materialism in previous studies [17,56,77]. Since the accuracy of memory on events written might influence manipulation magnitude, we controlled for accuracy of memory in the data analyses.

## 3. Results

### 3.1. Preliminary Analyses

After controlling for accuracy of memories, personal income, and socioeconomic status, consistent with our hypothesis, there was a significant condition effect. Participants in the experimental condition reported significantly higher scores on mortality salience (*M* = 1.57, *SD* = 2.31) and on gratitude (*M* = 3.56, *SD* = 1.17) than the scores of participants in the control condition on mortality salience (*M* = 0.33, *SD* = 1.20; *b* = 1.24, *SE* = 0.26, *t* = 4.75, *p* < 0.001, 95% CI (0.73, 1.76)), and on gratitude (*M* = 3.11, *SD* = 1.15; *b* = 0.44, *SE* = 0.17, *t* = 2.62, *p* < 0.01, 95% CI (0.11, 0.77)). There was also a significant difference in materialism in the two conditions. Compared with those in the control condition (*M* = 3.13, *SD* = 0.53), participants in the experimental condition expressed a significantly lower level of materialism (*M* = 2.98, *SD* = 0.63; *b* = −0.17, *SE* = 0.08, *t* = −2.09, *p* = 0.038, 95% CI (−0.34, −0.01)) (see Table 1). Correlations among the studies variables are reported in Table S1 in the Supplementary Materials.

### 3.2. Overview of Data Analysis

We first conducted separate analyses to test the mediating role of mortality salience and gratitude. We conducted simple mediation analyses using the PROCESS macro version 3.4.1 (Model 4) [78] with the condition as the independent variable, mortality salience (or gratitude) as the mediator, and materialism as the dependent variable while controlling for the accuracy of memory, personal income, and socioeconomic status. Next, we conducted an analysis considering the mediating roles of both mortality salience and gratitude simultaneously to further examine the dual-existing mechanisms. To do so, a parallel mediation analysis (model 4) with two mediators in PROCESS version 3.4.1 [79] was conducted.

#### 3.2.1. The Mediating Role of Mortality Salience

**Figure 1:**
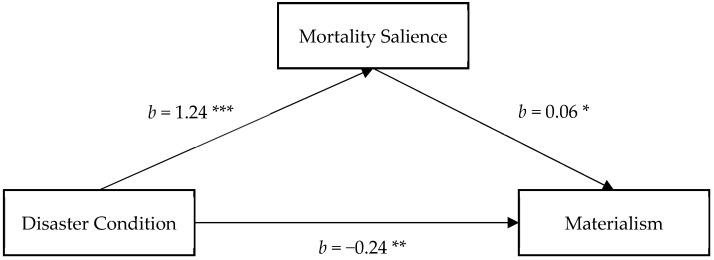
Mortality salience as a mediator of the relationship between the condition and materialism. Note: Reported values are unstandardized regression coefficients and standardized indirect effects with bootstrapped 95% confidence interval, *** *p* < 0.001, ** *p* < 0.01, * *p* < 0.05.

The results revealed a significant disaster manipulation effect in predicting mortality salience, in which participants in the disaster manipulation condition reported a higher level of mortality salience than did those in the control condition (*b* = 1.24, *SE* = 0.26, *p* < 0.001, 95% CI = (0.72, 1.76)). A higher level of mortality salience was related to more materialism (*b* = 0.06, *SE* = 0.02, *p* = 0.014, 95% CI = (0.01, 0.10)). However, the disaster manipulation showed significant negative effect on materialism, −0.17. After taking the effect of mortality salience into account, the effect of disaster manipulation on materialism was great (*b* = −0.24, *SE* = 0.09, *p* = 0.006, 95% CI = (−0.41, −0.07)). Importantly, the indirect effect of disaster manipulation on materialism via mortality salience was significant, the indirect effect estimate = 0.07, 95% CI = (0.01, 0.15) (Figure 1), showing that mortality salience was a significant mediator on increasing materialism.

#### 3.2.2. The Mediating Role of Gratitude

**Figure 2:**
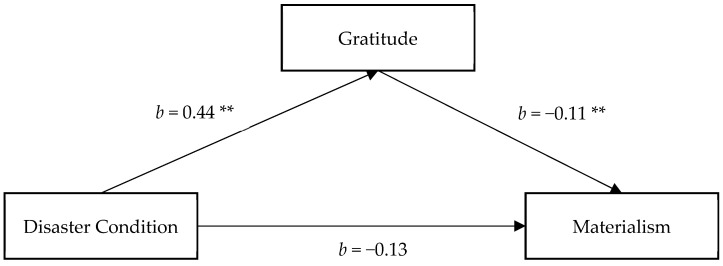
Gratitude as mediator of the relationship between condition and materialism. Note: Reported values are unstandardized regression coefficients and standardized indirect effects with bootstrapped 95% confidence interval, ** *p* < 0.01.

As shown in Figure 2, there was a significant disaster manipulation effect in predicting gratitude, as participants in the experimental condition reported a higher level of gratitude than control group (*b* = 0.44, *SE* = 0.17, *p* = 0.009, 95% CI = (0.11, 0.77)). A higher level of gratitude was associated with a lower level of materialism (*b* = −0.11, *SE* = 0.04, *p* = 0.003, 95% CI = (−0.18, −0.04)). The disaster manipulation showed significant effect on materialism, −0.17. After taking the effect of gratitude into account, the effect of disaster manipulation on materialism was weakened (*b* = −0.13, *SE* = 0.08, *p* = 0.132, 95% CI = (−0.29, 0.04)). Importantly, the indirect effect of disaster manipulation on materialism via mortality salience was significant, the indirect effect estimate = −0.05, 95% CI = (−0.10, −0.01), showing that gratitude was a significant mediator on reducing materialism.

#### 3.2.3. The Mediating Roles of Both Mortality Salience and Gratitude

**Figure 3:**
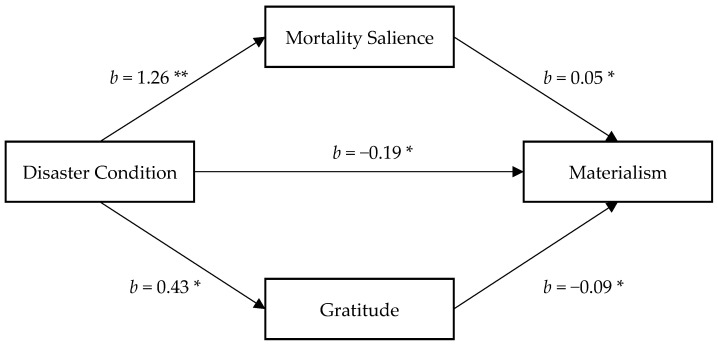
Mortality salience and gratitude as mediator of the relationship between condition and materialism. Note. Reported are unstandardized regression coefficients and standardized indirect effect with bootstrapped 95% confidence interval, ** *p* < 0.001, * *p* < 0.05.

With considering the mediating roles of both morality salience and gratitude, we received results to support both underlying mechanisms. In the parallel mediation model, being in the experimental condition was associated with a higher level of mortality salience (*b* = 1.26, *SE* = 0.26, *p* < 0.001, 95% CI = (0.74, 1.78)) and more gratitude (*b* = 0.43, *SE* = 0.17, *p* = 0.011, 95% CI = (0.10, 0.77)) simultaneously compared to the control condition. More mortality salience was related to more materialism (*b* = 0.05, *SE* = 0.02, *p* = 0.047, 95% CI = (0.001, 0.09)), whereas more gratitude was related with less materialism (*b* = −0.09, *SE* = 0.04, *p* = 0.010, 95% CI = (−0.16, −0.02)). The disaster manipulation showed a significant effect on materialism, −0.17. After taking into account the disaster manipulation’s indirect effect through mortality salience and gratitude, the effect of disaster manipulation on materialism was significant (*b* = −0.19, *SE* = 0.09, *p* = 0.035, 95% CI = (−0.36, −0.01)). Moreover, the indirect effect through mortality salience showed a significant positive effect (0.06) with 95% CI = (0.005, 0.14) on materialism, whereas the indirect effect through gratitude showed a significant negative effect (−0.04) with 95% CI = (−0.10, −0.003) on materialism (Figure 3).

## 4. Discussion

By activating disaster-related thoughts through video clips in an online experiment, the current study investigated the effect of disasters on materialism and the mediating roles of mortality salience and gratitude underlying the influence. We found that when participants were reminded of disasters, they reported greater mortality salience and gratitude than did participants in the control condition. Importantly, both mortality salience and gratitude significantly mediated the influence of disasters on materialism with the former one strengthening materialism but the latter one weakening materialism.

Materialism has emerged as an increasingly important research topic and attracts scholars in various fields, including political science [80], social psychology [81] and consumer research [3]. Although previous studies have examined the relationship between disaster and materialism, their findings have been inconclusive [5,14,82]. In this study, we posited and examined a dual mechanism underlying the influence of disaster and materialism. Cozzolino [83] proposed the dual-existential-system model to explore the sociopsychological outcomes of mortality salience. In particular, Cozzolino [83] argued that thoughts about death could activate two distinct systems, namely the abstract existential system and the specific existential system. The subtle feeling of mortality awareness may lead to anxiety and therefore a greater level of materialism, whereas a specific reflection on one’s own death may lead to a more prosocial donation (i.e., less materialistic behaviors). In the current study, mortality salience might have been relatively abstract to our participants who had thus far survived the pandemic; the feeling of gratitude, however, may have been more specific to each person because of their different life experiences. Our dual-mediator model illustrates the complex relationship between disaster and materialism. We found a significant causal relationship between disaster experience and mortality salience. Specifically, focusing on disaster triggered more mortality salience and mortality salience was associated with a higher level of materialism. Focusing on disaster also triggered more gratitude, and a higher level of gratitude was associated with a lower level of materialism. In our study, the association between disaster experience and materialism was negative, suggesting that gratitude may play an important role in the response to the COVID-19 pandemic.

Most previous studies on disaster have focused on the role of mortality salience. Fewer studies have highlighted the role of gratitude. Our study demonstrates the benefits of feeling gratitude after a disaster. In particular, feelings of gratitude induced by a disaster experience may lead individuals to consider more positive ways to cope with the effects of disaster [35,36] and reduce the importance of money and possessions [65]. Survivors of disasters who experienced relief and gratitude were shown to consider themselves as the lucky ones who had escaped death [84]. Just as money and possessions are pursued to obtain a sense of security, gratitude enables individuals to feel that they are supported by others and are thus secure and satisfied to some extent, explaining why participants in the experimental condition reported lower levels of materialism than did those in the control condition. For instance, Lambert et al. [85] found that the negative relationship between gratitude and materialism was mediated by a higher level of life satisfaction. The feeling of gratitude buffers the negative relationship between negative affect and materialism, and the relationship was weaker in individuals with high feelings of gratitude [86]. These findings suggest that interventions aiming at inducing feelings of gratitude after a disaster might buffer the anxiety and insecurity caused by the disaster and thereby promote better well-being [87].

The competing effects of mortality salience and gratitude have implications for theoretical and empirical exploration of needs satisfaction in the context of disasters. Maslow [88] proposed that fundamental needs must be satisfied before motivation can be increased. Thus, our results are partially consistent with Maslow’s theory of a hierarchy of needs. During a time of the disaster, individuals’ behaviors follow Maslow’s expectation that they will focus on the consumption of essential goods to reduce insecurity and cope with the fear of death. Their materialism is therefore strengthened at that time. After the disaster, however, internal motivations drive people to prioritize their psychological and self-fulfillment needs and lead them to experience more gratitude and engage in more prosocial behaviors. Further, Kasser and Ryan [81] differentiated intrinsic and extrinsic values and suggested that a balance between these values is critical when predicting harmful social and physical effects. In this way, gratitude might buffer the effects of pursuing materialism by triggering the pursuit of intrinsic goals or higher-order needs, such as self-expression and self-actualization [65,85], whereas materialism is more strongly associated with extrinsic goals and lower-order needs such as possessions and safety [89]. From the perspective of value orientation, the impact of disasters on people’s mortality salience and gratitude pulls people in opposite directions: mortality salience promotes valuing possessions and social status, whereas gratitude promotes valuing meaningful goals and activities. Future studies could investigate the relationship between disaster and materialism from the perspective of self-determination theory [90].

Our findings offer a new perspective on the mechanism of individuals’ emotional responses to disasters and their impacts on personal values during the COVID-19 pandemic. First, our findings suggest that a disaster experience could trigger a range of emotional responses simultaneously. The effects of disasters may depend on which emotions have been triggered. Adaptive emotions, such as gratitude, may help individuals to better cope with a disaster. Other emotions, such as anxiety caused by mortality salience, may worsen individuals’ experiences. Future studies could pay more attention to the network of emotions induced by disasters. Second, the study examined the mediating role of gratitude in the relationship between disasters and materialism. Other studies have also demonstrated the benefits of feeling gratitude during the COVID-19 pandemic. For instance, Jiang [91] found that, on days when individuals felt more gratitude, they reported better well-being and subjective health. Therefore, counsellors and psychologists, as well as educators, could provide helpful and adaptive coping strategies by focusing on inducing gratitude to alleviate the psychological distress caused by the pandemic.

We acknowledge some limitations of the current study. First, we relied on self-reported materialism. Future studies should adopt behavioral indexes of materialism, such as donations, to validate our findings. Second, we adopted an experimental design in which disaster experience was manipulated, which was only able to capture the immediate temporary effect of disaster experiences on materialism. A longitudinal study may better clarify the long-term relationships between disaster experience, materialism, mortality salience, and feelings of gratitude. Third, the priming materials of the current study focused on COVID-19; we did not examine whether the proposed model could be applied in other disaster contexts. Although the relationships among disaster, mortality salience, gratitude, and materialism were validated in multiple disaster settings, the proposed model has not been examined in another disaster context. Future studies should further validate the findings in other disaster-specific contexts. Fourth, because we relied on the study conducted in China, the generalization of findings could only be limited under the Chinese context. Because of the potential impacts of sociocultural factors on materialism [92], the findings should be further validated in different cultural samples. Fifth, some measures in the current study, such as gratitude and accuracy of memory, were measured by a single item. These measures could be improved by having multiple items to increase their validities in future studies. Sixth, we adopted the definition of Richins and Dawson (1992) [3] in this study, which defined materialism to be a value that reflects how one believes that possessions and material goods are important in one’s life. However, materialism may be a multidimensional construct. For instance, materialism is also suggested to signify one’s success [1]. According to the socioemotional selectivity theory [40], striving for success may not be consistent with individuals’ goals when they experience a limited future time perspective, as it always takes time to achieve success. Accumulating more stuff and perceiving to have more possessions to be happier and more successful, which were the dimensions of materialism examined in the present study, may be more manifest than other dimensions of materialism in the context of disaster, as it is relevant to survival. Therefore, it may be possible that the effects of experiencing a disaster on materialism differ in different dimensions of materialism. Future studies should further clarify this speculation.

## 5. Conclusions

To conclude, we examined the effect of disasters on materialism and how this relationship is mediated by mortality salience and gratitude. We found that a disaster experience decreased materialism. In particular, disaster experience triggered higher levels of both mortality salience and gratitude simultaneously. Mortality salience was associated with a higher level of materialism, whereas gratitude was associated with a lower level of materialism. By clarifying these two mechanisms, our findings provide insight into the relationship between disasters and materialism.

## Figures and Tables

**Table 1:** Descriptive information of participants.

Variables	Experimental Condition (*n* = 86)	Control Condition (*n* = 107)	*t*-Test	*p*
Accuracy of Memory	4.17 (1.07)	4.30 (0.72)	−0.93	0.354
Personal Income	2.24 (1.32)	2.43 (1.33)	−0.97	0.334
Socioeconomic Status	2.91 (1.60)	2.75 (1.35)	0.74	0.462
Mortality Salience	1.57 (2.31)	0.33 (1.20)	4.79	0.000
Gratitude	3.56 (1.17)	3.11(1.15)	2.69	0.008
Materialism	2.98 (0.63)	3.14 (0.53)	−1.80	0.073

## Data Availability

The data that support the findings of this study are available from the authors upon reasonable request.

## Supplementary Materials

The following are available online at https://www.mdpi.com/article/10.3390/ijerph18168566/s1, Table S1: Correlations among the Studied Variables.
