# Supplementary material for: Do People Become More or Less Materialistic during Disasters? The Mediating Roles of Mortality Salience and Gratitude
Source: Int J Environ Res Public Health. 2021 Aug 13;18(16):8566. doi: 10.3390/ijerph18168566 (PMC8392249; doi:10.3390/ijerph18168566)
Supplement: Supplementary file 1 [file ijerph-18-08566-s001.zip › ijerph-1313637-supplementary.pdf]

Supplementary Materials

Table S1. Correlations among the Studied Variables.

	Variable	1	2	3	4	5	6
1	Mortality Salience	1	−0.100	0.108	−0.024	0.041	0.083
2	Gratitude	−0.100	1	−0.210 **	0.066	−0.087	0.105
3	Materialism	0.108	−0.210 **	1	−0.038	−0.200 **	−0.003
4	Accuracy of Memories	−0.024	0.066	−0.038	1	0.171 *	−0.162 *
5	Personal Income	0.041	−0.087	−0.200 **	0.171 *	1	0.084
6	Socioeconomic Status	0.083	0.105	−0.003	−0.162 *	0.084	1

Note: ** $p < 0.01$, * $p < 0.05$.
